# The bacterioplankton community composition and a host genotype dependent occurrence of taxa shape the *Daphnia magna* gut bacterial community

**DOI:** 10.1093/femsec/fiaa128

**Published:** 2020-06-23

**Authors:** Martijn Callens, Luc De Meester, Koenraad Muylaert, Shinjini Mukherjee, Ellen Decaestecker

**Affiliations:** Laboratory of Aquatic Biology, Department of Biology, University of Leuven – Campus KULAK, E. Sabbelaan 53, B-8500 Kortrijk, Belgium; CEFE, Univ Montpellier, CNRS, EPHE, IRD, Univ Paul Valéry Montpellier 3, Montpellier, France; Laboratory of Aquatic Ecology, Evolution and Conservation, University of Leuven, Charles Deberiotstraat 32, 3000 Leuven, Belgium; Institute of Biology, Freie Universität Berlin, Köning-Luise-Strasse 1–3, 14195 Berlin, Germany; Leibniz Institut für Gewasserökologie und Binnenfischerei (IGB), Müggelseedamm 310, 12587 Berlin, Germany; Laboratory of Aquatic Biology, Department of Biology, University of Leuven – Campus KULAK, E. Sabbelaan 53, B-8500 Kortrijk, Belgium; Laboratory of Aquatic Ecology, Evolution and Conservation, University of Leuven, Charles Deberiotstraat 32, 3000 Leuven, Belgium; Laboratory of Aquatic Biology, Department of Biology, University of Leuven – Campus KULAK, E. Sabbelaan 53, B-8500 Kortrijk, Belgium

**Keywords:** gut bacterial community, *Daphnia magna*, community assembly, colonization, bacterioplankton, host genotype

## Abstract

The assembly of host-associated bacterial communities is influenced by a multitude of biotic and abiotic factors. It is essential to gain insight in the impact and relative strength of these factors if we want to be able to predict the effects of environmental change on the assembly of host-associated bacterial communities, or deliberately modify them. The environmental pool of bacteria, from which the host is colonized, and the genetic background of the host are both considered to be important in determining the composition of host-associated bacterial communities. We experimentally assessed the relative importance of these two factors and their interaction on the composition of *Daphnia magna* gut bacterial communities. Bacterioplankton originating from natural ponds or a laboratory culture were used to inoculate germ-free *Daphnia* of different genotypes. We found that the composition of the environmental bacterial community has a major influence on the *Daphnia* gut bacterial community, both reflected by the presence or absence of specific taxa as well as by a correlation between abundances in the environment and on the host. Our data also indicate a consistent effect of host genotype on the occurrence of specific bacterial taxa in the gut of *Daphnia* over different environments.

## Teaser

The assembly of the *Daphnia magna* gut microbiota is strongly influenced by the composition of the environmental bacterioplankton community, and additionally structured by the host genetic background.

## INTRODUCTION

Numerous studies have shown that the health and fitness of animals can strongly depend on the composition of their associated bacterial communities. Multiple factors have been identified that influence the composition of host-associated bacterial communities, e.g. the pool of environmental bacteria, ecological interactions with other members of the microbiota, host genotype, diet and abiotic conditions (Smith *et al*. 2015; Wexler *et al*. 2016; Sprockett, Fukami and Relman 2018; Suzuki, Martins and Nachman 2019). These factors can thus have an indirect effect on the host's health and fitness, as their effect on the composition of host-associated bacterial communities can translate in changes in host phenotype. Community assembly is, however, a complex process of which the outcome depends on multiple deterministic drivers as well as on stochastic processes (Vega and Gore 2017; Macke *et al*. 2017a). For example, continuous high dispersal rates of a taxon could determine its persistence in the community through mass effects, while the identity of early colonizers—which can be more stochastic—could also significantly affect assembly through priority effects (Vass and Langenheder 2017). Obtaining insight into the relative strength and interaction between factors shaping the gut bacterial community composition is essential, as this can allow to better predict the potential impact of environmental change on host-associated bacterial communities or enable to manage or deliberately modify these communities to improve the host's health.

Over the past decade, *Daphnia magna* has emerged as an interesting model system for experimentally investigating the effects of environmental and genetic factors on host-associated bacterial community assembly (Qi *et al*. 2009; Freese and Schink 2011; Gorokhova *et al*. 2015; Macke *et al*. 2017a; Callens *et al*. 2018, Sullam *et al*. 2018; Frankel-Bricker *et al*. 2019; Mushegian *et al*. 2019). Its suitability for addressing these questions is mainly due to its background as an ecological model system (Miner *et al*. 2012), its fast clonal reproduction—allowing for large experiments with isogeneic individuals and a direct test of genotype-dependent responses—and the availability of methods for generating germ-free individuals (Peerakietkhajorn *et al*. 2015; Sison-Mangus, Mushegian and Ebert 2015; Callens *et al*. 2016).

Previous work has documented a significant impact of multiple environmental factors on the composition of *Daphnia*-associated bacterial communities: diet (Freese and Schink 2011; Macke *et al*. 2017a), the environmental pool of bacteria (Macke *et al*. 2017a; Callens *et al*. 2018; Mushegian *et al*. 2019), antibiotic exposure (Gorokhova *et al*. 2015; Callens *et al*. 2018) and temperature (Sullam *et al*. 2018; Frankel-Bricker *et al*. 2019). Several of these experiments also included different clonal lineages to simultaneously study the effect of host genotype on community composition. Sullam *et al*. (2018) found significant differences between different host clonal lineages, but no correlation between the composition of associated bacterial communities and geographical origin. Frankel-Brickel *et al*. (2019), on the other hand, report a significant effect of geographical origin but no differences between clonal lineages from a specific location. Sison-Mangus, Metzger and Ebert (2018) also observed an effect of host genotype (all originating from different geographical locations) and found this to be correlated with the resistance profile of *D. magna* against the parasitic bacterium *Pasteuria ramosa*. Macke *et al*. (2017a) similarly found a correlation between gut bacterial community composition and tolerance of *D. magna* genotypes to the toxic cyanobacterium *Microcystis aeruginosa*. They showed through microbiota transplant experiments that tolerance profiles between genotypes were shaped by their microbiota. Interestingly, the effect of the transplanted bacterial communities on the community composition in the recipient was only transient, with the influence of the receiving genotype becoming more dominant over time. Little is known about the mechanistic link between host genotype and the composition of associated bacterial communities in *Daphnia*, but Mushegian *et al*. (2019) showed that genetically determined differences in sediment browsing behavior of *D. magna* have a significant influence on the host-associated bacterial community composition through differences in uptake of sediment-associated bacteria.

Most experiments assessing the effect of environmental or genetic factors on the composition of host-associated bacterial communities were conducted using bacterial communities that occur in laboratory cultures. First, these can differ strongly from those encountered in the environments inhabited by natural *Daphnia* populations (Callens 2017). Second, differences in bacterial communities between cultures of *Daphnia* clones might confound genotype effects through intra-clone horizontal transmission of differentiated bacterial communities (so-called ‘legacy effect’; Rawls *et al*. 2006). All of the above-mentioned studies except Macke *et al*. (2017a) reporting an influence of host genotype on host-associated bacterial community composition did not account for this effect. It can, however, be controlled for by inoculation of germ-free individuals from each genotype with identical bacterial communities. We here investigated the assembly of gut bacterial communities in multiple *D. magna* genotypes, each exposed to identical and diverse bacterioplankton communities isolated from natural ponds or from a laboratory culture. We consistently re-inoculated the medium with these bacterioplankton communities throughout the experiment to prevent strong modifications of the bacterioplankton community through selective lab conditions or impacts by *Daphnia* (Macke *et al*. 2020). This enables us to disentangle the effect of environmental bacteria, as a source of taxa from which gut bacterial communities are assembled, and host genotype on the *Daphnia* gut bacterial community composition under controlled conditions while mimicking more diverse and natural settings.

## MATERIALS AND METHODS

### *Daphnia* genotypes

A total of five *D. magna* genotypes were used to investigate the influence of different bacterial environments and host genotype on the assembly of gut bacterial communities. All genotypes were isolated several years prior to this experiment and have thus been kept in laboratory cultures for multiple parthenogenetic generations. From different natural ponds and lakes, three genotypes, KNO15.04, OM2NF8 and BSW7 were isolated and their associated bacterial communities have already been investigated in other studies (Callens *et al*. 2016; Macke *et al*. 2017a). Genotype KNO15.04 originates from a small (350m²) fishless mesotrophic pond in Knokke, at the Belgian coast (51°20′05.62″ N, 03°20′53.63″ E). OM2NF8 originates from a 3.7 ha eutrophic inland pond containing fish, located in Heverlee, Belgium (Oude meren, Abdij van 't Park; 50°51′47.82″ N, 04°43′05.16″ E). BSW7 originates from Bysjön lake in Sweden (59°48′37.25″ N, 12°20′50.96″ E). Additionally, two other genotypes, NIES and F-clone, are standard genotypes used in ecotoxicological testing (Barata *et al*. 2017). For the NIES genotype, the effect of inoculating disturbed bacterial communities on the assembly of *Daphnia*-associated bacterial communities and on host performance is described in Callens *et al*. (2018).

### Cultivation of axenic *Chlorella*

Sterile cultures of *Chlorella vulgaris* were started by inoculating a small amount of algae from a sterile stock culture kept on WC-agar [adapted from Guillard and Lorenzen (1972) with the addition of 1.5% agar] into an Erlenmeyer flask containing WC medium. When there was visible growth of microalgae in the flask, the culture was inoculated in a bottle containing 2 L of WC medium and bottles were closed with a sterile cap. Cultures were grown under constant aeriation and stirring at 22°C ± 1°C under fluorescent light (120 µmoL/m^2^/s) at 16:8 h light: dark. Algae were harvested during exponential growth by centrifugation and resuspended in sterile ADaM medium. After harvesting, each batch of algae was checked for bacterial contamination by investigating DAPI-stained samples under a fluorescence microscope at x1000 magnification and by plating on nutrient agar. Aliquots of sterile *Chlorella* were stored at −20°C until further use.

### Generation of germ-free *Daphnia*

All *Daphnia* genotypes were kept for three generations under identical circumstances in 0.5 L jars filled with ADaM on a diet of saturating amounts of *Chlorella*. To stimulate egg production, 125 µm filtered water from a natural pond was regularly added to the cultures. For each genotype, 150 parthenogenetic eggs were isolated by opening the brood pouch of the mother with two fine needles and transferring the released eggs to a petri dish using a micropipette. The eggs were subsequently disinfected in batches of 50 by exposing them for 10 min to 0.01% peracetic acid (Sigma-Aldrich, Munich, Germany) as described in Callens *et al*. (2016). To hatch the disinfected eggs, each batch was incubated for 72 h in a petri dish containing filtered ADaM. Hatched individuals (germ-free *Daphnia*) were subsequently used to set up the inoculation experiment (see below).

### Bacterial inocula

#### Pond water

Pond water containing natural bacterioplankton communities was used to inoculate the experimental units. This pond water was collected the day before inoculation from three ecologically very different ponds. The first pond (50°48′58.63″ N, 03°15′14.44″ E, annotated as **‘*Daphnia* pond’**) is a shallow pond (max. depth around 0.5 m) located in a city park. This pond contains no fish but harbors a population of both *D. magna* and *D. pulex*. A second pond (50°47′54.56″ N, 03°15′14.44″ E, annotated as **‘fish pond’**) is a shallow pond (max. depth around 1 m) located in a small patch of woodland. It contains fish and is characterized by abundant macrophyte growth. Cladocerans belonging to the genus *Daphnia* were not observed during the sampling period (late autumn). A third pond (50°48′28.90″ N, 03°17′34.00″ E, annotated as **‘reservoir’**) is a shallow (max. depth around 1 m) artificial water reservoir containing fish and is characterized by frequent algal blooms in summer. Cladocerans belonging to the genus *Daphnia* were not observed at the time of sampling. Water originating from the surface of each pond was filtered over a 180 µm mesh and collected in a 2 L plastic bottle. The collected pond water was placed for 24 h at 4°C to allow for the sedimentation of phytoplankton and other small particles.

#### Hay extract

In addition to using three natural bacterioplankton communities as bacterial inoculum, we also made an additional inoculation using bacteria grown on hay extract. Hay extract is sometimes used as a supplement to *Daphnia* cultures, and has been found to increase growth and reproduction in the lab (Luc De Meester, personal observations). To prepare the hay extract based bacterial inoculum, 2 L of ADaM medium containing 50 g of hay was autoclaved for 20 min at 121°C. After autoclaving, the extract was filtered over a 180 µm mesh to remove the hay from the medium. The hay extract was subsequently inoculated with 10 mL of medium originating from a healthy stock culture of *Daphnia*. This inoculated extract was placed for 3 days at 22°C with aeration and stirring to allow for bacterial growth. This single culture was used to inoculate experimental units throughout the experiment. The culture was kept at 4°C between subsequent inoculations.

### Inoculation experiment

**Figure 1.**
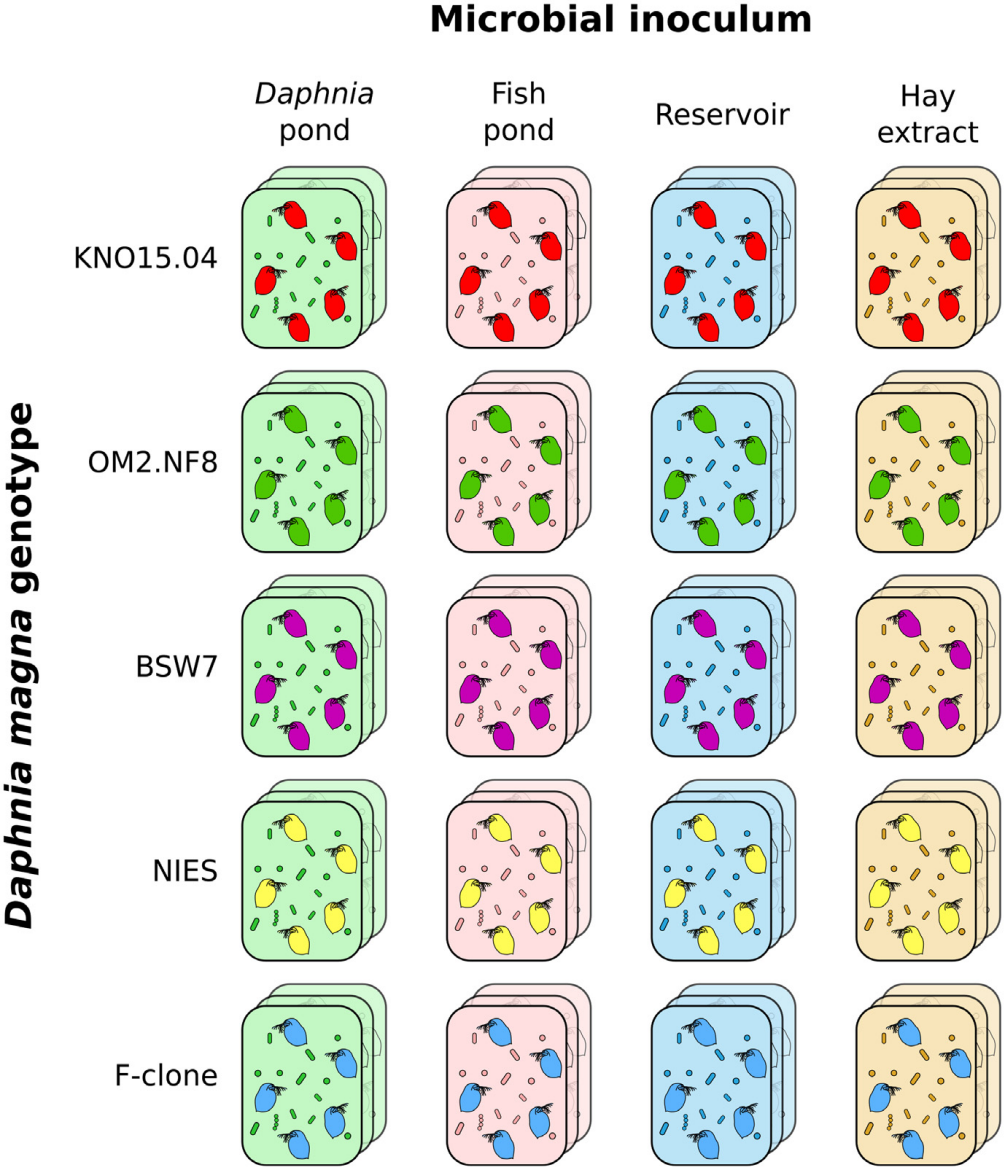
Experimental setup of the inoculation experiment. A total of five individuals of germ-free *Daphnia* belonging to one of five different genotypes were inoculated with one of four different bacterial communities originating from either natural ponds or from a lab culture of bacteria grown on hay extract. *Daphnia* gut bacterial communities were allowed to establish for 10 days, after which the *Daphnia* guts were dissected to characterize the gut bacterial community. Bacterial communities in the medium of the experimental units were simultaneously characterized. Each treatment was set up in triplicate.

Experimental units containing different bacterial inocula were set up by filling glass jars with 45 mL of 0.22 µm filtered ADaM and adding 5 mL of pond water collected the day before. Experimental units containing bacterial inocula from hay extract contained 49.5 mL filtered ADaM to which 0.5 mL of hay extract was added. The amount of hay extract was kept low because of high bacterial densities in hay extract. Total bacterial cell numbers in different inoculation treatments might still have shown considerable variation, as it was not possible for us to measure and standardize this during the experiment. For each genotype, hatched germ-free *Daphnia* from all three disinfected egg batches were pooled in a single petri-dish. A total of five individuals were randomly picked from the pool and placed in a single experimental unit. Each host genotype/bacterial inoculum combination was replicated three times, which resulted in a total of 60 experimental units (5 host genotypes × 4 bacterial inocula × 3 replicates, Fig. 1). Every other day, half of the medium was replaced with fresh medium having the same ratio of ADaM/bacterial inoculum as mentioned above. Bacterial inocula from pond water were collected each time the day before use in the experiment. After 4 days, the amount of medium was increased to a final volume of 150 mL by adding 100 mL of the appropriate ADaM/bacterioplankton mixture to each experimental unit. Each experimental unit was fed daily with 2 × 10^5^ cell/mL of axenic *Chlorella*. *Daphnia* were kept for 10 days in the experimental units to allow them to reach reproductive age and for the establishment of a mature gut bacterial community.

### Sampling of bacterial communities

On day 10, all surviving individuals from each experimental unit were kept for 3 h in 15 mL falcon tubes filled with filtered ADaM and 1.5 g/L of cellulose microparticles (20 µm cellulose microcrystalline powder, Sigma-Aldrich). The addition of microparticles allowed for the removal of transient bacteria as gut content is filled with the sterile cellulose microparticles through grazing (gut clearance). For each replicate, the guts of three individuals were dissected and frozen at −80°C until further processing. Bacterial communities in the medium were collected from each experimental unit at the end of the experiment by passing 50 mL of the medium over a 0.22 µm nitrocellulose filter (Millipore, Burlington, Massachusetts, USA); filters were stored at −80°C until further processing.

### Sequencing library preparation

After DNA extraction from both the gut samples and the filters containing bacterioplankton communities (following Callens *et al*. 2016), the full length 16S rRNA gene was amplified in all samples using primers 27F and 1492R (94°C–30 s; 50°C–45 s; 68°C–90 s; 30 cycles) using a high-fidelity Pfx polymerase (Life technologies, Waltham, Massachusetts, USA). PCR products were purified using the QIAquick PCR purification kit (Qiagen, Hilden, Germany). To obtain dual-index amplicons of the V4 region, a second amplification was performed on 5 µL of PCR product using primers 515F (Kozich *et al*. 2013) and a slightly modified version of primer 806R to increase detection of SAR11 bacterioplankton (Apprill *et al*. 2015) for 30 cycles (94°C–30 s; 55°C–30 s; 68°C–60 s). Both primers contained an Illumina adapter and an 8-nt barcode at the 5′-end. For each sample, PCRs were performed in triplicate, pooled and gel purified using the QIAquick gel extraction kit (Qiagen). An equimolar library was prepared by normalizing amplicon concentrations with a SequalPrep Normalization Plate (Applied Biosystems, Waltham, Massachusetts, USA) and subsequent pooling. Amplicons were sequenced using a v2 PE500 kit with custom primers (Kozich *et al*. 2013) on the Illumina Miseq platform (KU Leuven Genomics Core, Leuven, Belgium), producing 2 × 250-nt paired-end reads.

Sequence reads were processed using R 3.3.2 (R Core Team 2019) following Callahan *et al*. (2016a). Sequences were trimmed (the first 10 nucleotides and from the position at which the mean quality score dropped below Q25 onward) and filtered (maximum of two expected errors per read) on paired ends jointly. Sequence variants were inferred using DADA2 (Callahan *et al*. 2016b). Chimeras were subsequently removed from the dataset. Taxonomy was assigned with a naïve Bayesian classifier using the Greengenes 13.8 training set. OTU's with no taxonomic assignment at phylum level or which were assigned as mitochondria were subsequently removed from the dataset. For some taxa of particular interest an additional taxonomic assignment was done by performing a nucleotide BLAST against the NCBI 16S ribosomal RNA database. After filtering, a total of 4642 557 reads were obtained, with a mean of 38 688 reads per sample and most samples having more than 10 000 reads (only two exceptions with 758 and 2821 reads). A neighbor joining phylogenetic tree was constructed and was used as a starting point for fitting a GTR+G+I maximum likelihood tree. Closely related OTU's were binned by applying tip agglomeration at a depth of 0.1 on the phylogenetic tree. OTU's for which the mean relative abundance over all the samples was below 0.1% were removed from the analysis.

### Statistical analysis of bacterial communities

To validate our approach of considering inoculation source as a categorical variable in the statistical analysis, we verified whether the composition of bacterial communities in the medium of the experimental units was differentiated according to their inoculation source. A principal coordinate analysis was performed on the Bray–Curtis dissimilarity matrix for all medium bacterial communities using the *ordinate* function in phyloseq (McMurdie and Holmes 2013). The resulting ordination axes were subsequently used in a partitioning clustering analysis (k-means clustering). The optimal number of clusters was determined by gap statistic values, which are obtained by comparing the total intracluster variation for different values of *k* with their expected values under a distribution with no obvious clustering. Gap statistic values from 100 bootstrap samples were obtained using the *fviz_nbclust* function in the factoextra package. Samples were clustered using k-means clustering with the calculated optimal number of clusters (*k* = 4) on 25 random initial sets.

To test for differences in α-diversity metrics between treatments, all samples were rarified to a depth of 10 000 reads. Two gut samples having a lower number of reads were removed from this analysis. Species richness, Shannon diversity and Shannon evenness were calculated for each sample. For gut bacterial communities, a two-way ANOVA was performed to test for differences in diversity parameters dependent on inoculation source, host genotype and their interaction. Pairwise differences between inoculation treatments were assessed using a post-hoc Tukey's HSD test. To test for differences in species richness in the bacterioplankton dependent on inoculation treatment, pairwise comparisons were performed using a one-way ANOVA followed by a post-hoc Tukey's HSD test. Correlation between species richness in the bacterioplankton and the gut bacterial community was determined using a linear regression.

The effects of inoculation source, host genotype and their interaction on the composition of gut bacterial communities were analyzed by permutational ANOVA (Anderson 2001) on both quantitative (Bray–Curtis dissimilarity and weighted unifrac) and qualitative (Jaccard and unweighted unifrac) distance matrices with 9999 permutations using the *adonis* function of the R package *vegan* v2.5 (Oksanen *et al*. 2019). To assess the relative contribution of the inoculum and host genotype on the gut bacterial community assembly we used a variation partitioning approach (Peres-Neto *et al*. 2006). Variation partitioning was performed for both Hellinger transformed counts and presence-absence data of the gut bacterial communities including both inoculation source and host genotype as variables (*varpart* function in vegan). Significance of each variable was assessed using the *rda* and *anova.cca* functions. In addition to testing differences in β-diversity between treatments on all inoculation treatments simultaneously, we did a separate analysis including only treatments inoculated with natural bacterioplankton communities. We did this because the importance of the bacterioplankton in shaping the gut bacterial community composition could be overestimated by including the artificial hay extract treatment in which the inoculum had a highly divergent composition compared to natural habitats. The effect of host genotype on the gut bacterial community composition was also additionally assessed for each inoculation treatment separately, as variation in gut bacterial community composition caused by the inoculum could potentially mask more subtle effects of the host genotype.

To identify bacterial taxa that have the largest contribution to the differentiation of the gut bacterial community composition according to inoculation source or host genotype, we performed a random forest analysis using OTU's as predictors and inoculation source or host genotype as response variable using the *randomForest* package (Liaw and Wiener 2002) with 100 classification trees.

Differential abundances of bacterial taxa in the *Daphnia* gut between different inoculation sources or host genotypes were tested using DeSeq2 (Love, Huber and Anders 2014). Because rare taxa often have a high number of zero counts, they can appear significantly differentially abundant by chance. To account for this issue, we performed 1000 permutations randomizing the assignment of the focal treatment to the samples. The fraction of permutations where a taxon is significantly differentially abundant indicates the potential importance of the issue for this specific taxon.

Significance of correlations between relative abundances of single OTU's in the medium and gut were assessed with a non-parametric test using Kendall's rank correlation coefficient. Only samples where the medium or the gut contained the focal OTU were included in the analysis to remove any artifacts caused by double zero values. A Bonferroni correction for multiple testing was applied on the obtained p-values before assessing the significance of each correlation.

## RESULTS

### Bacterial communities in the medium of different inoculation treatments

**Figure 2.**
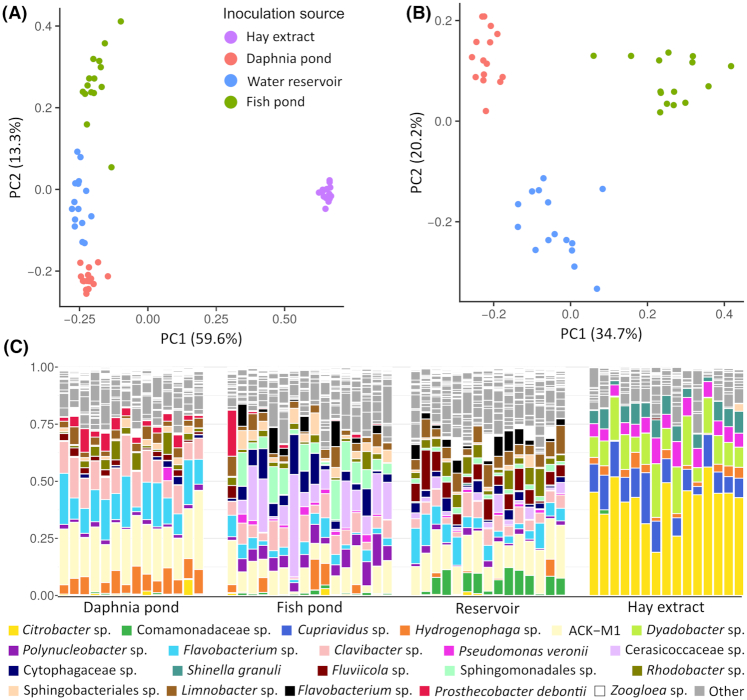
Composition of bacterial communities in the medium of different inoculation treatments. **(A)** PCoA based on Bray–Curtis dissimilarity for the three different inocula from natural habitats and the hay extract. **(B)** PCoA based on Bray–Curtis dissimilarity when only the three inocula from natural habitats are included. **(C)** Relative abundance of OTU's in all medium samples for different experimental units. For clarity, only OTU's which were either the most abundant taxon in at least one sample or OTU's that where in the three most abundant taxa in at least two samples are color-coded.

Based on the gap statistic, bacterial communities in the medium of the four different inocula could be grouped into four distinct clusters. K-means clustering resulted in clusters consisting exclusively of samples from the same treatment (i.e. inoculated from a single source). Ordination shows that bacterial communities in the medium inoculated with hay extract were well differentiated from all other treatments (Fig. 2A). Ordination of the treatments inoculated with bacterioplankton from a natural source further shows that three well-differentiated clusters were formed, reflecting consistent differences in community composition over the different inocula (Fig. 2B). These results support the validity of considering inoculation source as a categorical variable in statistical testing.

Overall, bacterial communities in the medium showed high variation in species richness between samples and treatments (ranging between 19 and 87 taxa). Bacterial communities in the medium of the hay extract treatment had a lower species richness than treatments inoculated with bacterioplankton from natural ponds (24.1 ± 2.9 taxa; *p*-adj. < 0.05). The fish pond inoculum had a lower species richness than the water reservoir (53.1 ± 12.2 and 65.1 ± 11.4 taxa, respectively; *p*-adj. < 0.05). Species richness in the medium inoculated with *Daphnia* pond bacterioplankton (62.3 ± 14.0 taxa) was not significantly different from species richness in the medium inoculated with fish pond or reservoir bacterioplankton.

In all samples of the hay extract treatments, bacterial communities in the medium were dominated by *Citrobacter* sp. (41.4 ± 9.8%). Other abundant taxa were *Dyadobacter* sp. (13.5 ± 5.5%) and *Cupriavidus* sp. (11.6 ± 4.4%, Fig. 2C). Samples inoculated with bacterioplankton from natural habitats were more similar to each other and well differentiated from the hay extract (Fig. 2A), mainly due to the shared occurrence of bacteria belonging to the ACK-M1 cluster, *Polynucleobacter* sp., *Flavobacterium* sp. and *Clavibacter* sp. In samples inoculated from the *Daphnia* pond, a large fraction of the bacterial community in the medium was comprised of a member of the actinobacterial ACK-M1 cluster (22.3 ± 4.7%), *Flavobacterium* sp. (13.7 ± 5.8%) and *Clavibacter* sp. (11.9 ± 3.6%). In medium inoculated with fish pond bacterioplankton, *Cerasicoccaceae*sp. was overall the most abundant taxon (13.5 ± 11.4%), but its abundance showed high variability between samples (ranging between 9.1 and 44.7%). Other abundant taxa, such as ACK-M1 sp. (9.7 ± 4.1%) and *Sphingomonadales*sp. (8.4 ± 5.3%) also showed a high degree of variability between samples within this treatment. In medium inoculated with bacterioplankton from the reservoir, a member of the ACK-M1 cluster was the overall most abundant taxon (15 ± 3.8%). However, in some experimental units the most abundant taxa were *Flavobacterium* sp. (7.2 ± 4.2%), *Fluviicola* sp. (7.0 ± 3.8%) or *Comamonadaceae*sp. (6.3 ± 4.3%).

### Composition of the *Daphnia* gut bacterial communities

**Figure 3.**
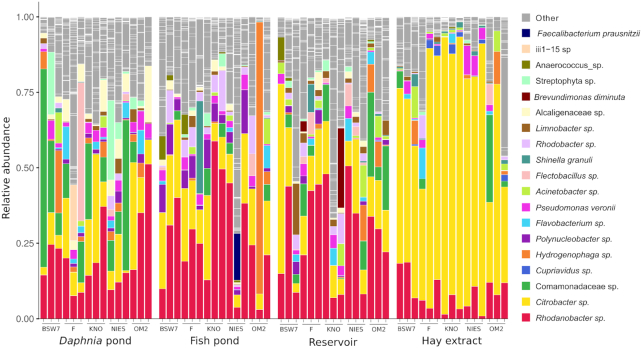
Composition of *Daphnia* gut bacterial communities. Relative abundance of OTUs in all gut samples for different inoculation treatments and host genotypes. For clarity, only OTUs which were either the most abundant taxon in at least one sample or OTUs that where in the three most abundant taxa in at least two samples are color-coded.

In the *Daphnia* gut, *Citrobacter* sp. and *Rhodanobacter* sp. were commonly found to be the most abundant taxa (Fig. 3). However, there were pronounced differences in the relative abundances of these taxa, both between and within inoculation treatments. Overall, samples inoculated with hay extract showed a higher abundance of *Citrobacter* sp. (65.6 ± 21.8%, with a relative abundance of >90% in some samples), while the abundance of *Rhodanobacter* sp. was lower (8.4 ± 5.6%). On the contrary, samples inoculated from the *Daphnia* pond, fish pond or reservoir showed the inverse pattern with an overall higher abundance of *Rhodanobacter* sp. (20.6 ± 12.2%, 27.4 ± 16.7% and 28.0 ± 16.0%, respectively) and an overall lower abundance of *Citrobacter* sp. (12.6 ± 10.9%, 16.6 ± 8.2% and 16.3 ± 14.1%, respectively).

In samples inoculated with *Daphnia* pond bacterioplankton, *Comamonadaceae*sp. (100% identity to *Limnohabitans*sp. in NCBI database) and *Alcaligenaceae*sp. commonly made up a large proportion of the gut bacterial community (6.6 ± 8.5% and 4.7 ± 4.9%, respectively), while these were less common in other inoculation treatments. Several generally rare taxa also reached a high abundance in single samples: one sample contained 45.9% *Flectobacillus* sp., two samples contained >20% *Streptophyta*sp. and in one sample three members of the Acidobacterial order iii1–15 compromised together 41.6% of the community.

Gut bacterial communities in treatments inoculated with fish pond bacterioplankton had a noticeable higher abundance of *Polynucleobacter* sp. (5.2 ± 4.3%) and *Rhodobacter* sp. (1.9 ± 2.7%). *Shinella granuli* and *Faecalibacterium prausnitzii* also reached high abundances in two separate samples (30.7% and 15.5%, respectively), and the gut bacterial community in one sample was also almost completely comprised of *Hydrogenophaga* sp. (90.1%).

*Daphnia* gut bacterial communities inoculated from the reservoir contained an overall higher proportion of *Limnobacter* sp. (2.9 ± 4.1%). *Brevundimonas diminuta* reached a high abundance in one sample within this treatment (26.5%).

### Influence of inoculum on gut bacterial community composition

**Figure 4.**
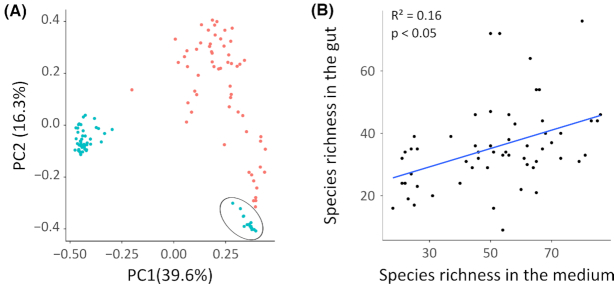
Comparison of bacterial communities in the *Daphnia* gut and medium. **(A)** PCoA based on Bray–Curtis dissimilarity including both bacterial communities in the gut (red) and bacterial communities in the medium (blue). Bacterial communities in the medium of the hay extract treatment are encircled. **(B)** Correlation between species richness in the gut and medium bacterial communities.

In treatments where natural bacterioplankton communities were used for inoculation, the community composition of the gut bacterial community was clearly differentiated from the bacterial community composition of the medium in which they were kept. The exception was the hay extract treatment, for which the medium and gut bacterial communities were more similar to each other. This was due to a high abundance of *Citrobacter* sp. in both the gut and the medium, and to an overall relatively high fraction of taxa that were shared between the medium and the gut bacterial communities in this treatment (Fig. 4A).

There was a significant effect of inoculation treatment on all α-diversity metrics in the gut bacterial communities (species richness, Shannon diversity and Shannon evenness) (Table 1). This pattern is mainly driven by a significantly lower Shannon diversity and evenness in the hay extract treatment compared to all other treatments (*P* < 0.05 for all post-hoc pairwise comparisons). Species richness only significantly differed between the gut bacterial communities inoculated with hay extract and fish pond bacterioplankton (pairwise post-hoc comparison: *P* < 0.05). No significant differences were found in the gut bacterial community α-diversity parameters between treatments inoculated with natural bacterioplankton communities. A significant positive correlation was found between species richness in the gut and medium (Fig. 4B; *P* < 0.05; R^2^ = 0.16) and between Shannon diversity in the gut and medium (*P* < 0.05; R^2^ = 0.14).

**Table 1.** Results for statistical tests assessing the effect of inoculation source, host genotype and their interaction on alpha-diversity (species richness, Shannon diversity and Shannon evenness), quantitative beta-diversity (Bray–Curtis dissimilarity and weighted unifrac distance) and quantitative beta-diversity metrics (Jaccard distance and unweighted unifrac distance) and results for variation partitioning analysis assessing the relative contribution of inoculation source and host genotype to differences in community composition. Tests were performed including all inoculation treatments or including only treatments that were inoculated with natural bacterioplankton communities.

	All inoculation treatments	Inoculated with natural bacterioplankton
**α-diversity measures (ANOVA)**		
**Species richness**		
Inoculation source	F_(3,38)_ = 3.725; *P* = 0.0192*	F_(2, 29)_ = 0.890; *P* = 0.422
Host genotype	F_(4, 38)_ = 1.038; *P* = 0.4003	F_(4,29)_ = 1.529; *P* = 0.220
Inoculation source × host genotype	F_(12, 38)_ = 0.655; *P* = 0.7819	F_(8, 29)_ = 0.366; *P* = 0.935
**Shannon diversity**		
Inoculation source	F_(3, 38)_ = 4.638; *P* = 0.0074*	F_(2, 29)_ = 0.050; *P* = 0.951
Host genotype	F_(4, 38)_ = 0.446; *P* = 0.7749	F_(4, 29)_ = 0.813; *P* = 0.527
Inoculation source × host genotype	F_(12, 38)_ = 0.763; *P* = 0.6828	F_(8, 29)_ = 0.497; *P* = 0.848
**Shannon evenness**		
Inoculation source	F_(3, 38)_ = 5.543; *P* = 0.0029*	F_(2, 29)_ = 0.330; *P* = 0.721
Host genotype	F_(4, 38)_ = 0.483; *P* = 0.7482	F_(4, 29)_ = 0.099; *P* = 0.982
Inoculation source × host genotype	F_(12, 38)_ = 0.784; *P* = 0.6632	F_(8, 29)_ = 0.523; *P* = 0.829
**Quantitative β-diversity measures (permutational ANOVA)**		
**Bray-Curtis dissimilarity**		
Inoculation source	F_(3, 59)_ = 7.223; *P* = 0.0001*	F_(2, 44)_ = 2.126; *P* = 0.0006*
Host genotype	F_(4, 59)_ = 0.921; *p* = 0.5887	F_(4, 44)_ = 0.821; *P* = 0.8629
Inoculation source × host genotype	F_(12, 59)_ = 1.145; *P* = 0.1724	F_(8, 44)_ = 1.117; *P* = 0.1803
**Weighted unifrac distance**		
Inoculation source	F_(3, 59)_ = 4.791; *P* = 0.0001*	F_(2, 44)_ = 1.823; *P* = 0.0138*
Host genotype	F_(4, 59)_ = 1.142; *P* = 0.2562	F_(4, 44)_ = 1.092; *P* = 0.3073
Inoculation source × host genotype	F_(12, 59)_ = 1.130; *P* = 0.1914	F_(8, 44)_ = 1.062; *P* = 0.3312
**Qualitative β-diversity measures (permutational ANOVA)**		
**Jaccard distance**		
Inoculation source	F_(3, 59)_ = 2.604; *P* = 0.0001*	F_(2, 44)_ = 2.143; *P* = 0.0001*
Host genotype	F_(4, 59)_ = 1.120; *P* = 0.0945	F_(4, 44)_ = 1.180; *P* = 0.0190*
Inoculation source × host genotype	F_(12, 59)_ = 1.016; *P* = 0.3759	F_(8, 44)_ = 1.00; *P* = 0.4751
**Unweighted unifrac distance**		
Inoculation source	F_(3, 59)_ = 2.360; *P* = 0.0001*	F_(2, 44)_ = 1.853; *P* = 0.0001*
Host genotype	F_(4, 59)_ = 1.268; *P* = 0.0158*	F_(4, 44)_ = 1.285; *P* = 0.0052*
Inoculation source × host genotype	F_(12, 59)_ = 1.015; *P* = 0.4006	F_(8, 44)_ = 0.949; *P* = 0.7524
**Variation partitioning analysis**		
**Hellinger transformed abundances**		
Inoculation source	30.6%; *P* = 0.001*	4.4%; *P* = 0.001*
Host genotype	n.s.	n.s.
**Presence-absence**		
Inoculation source	6.2%; *P* = 0.001*	4.2%; *P* = 0.001*
Host genotype	n.s.	1.3%; *P* = 0.016*

There was a consistent effect of the inoculation source on *Daphnia* gut bacterial community composition for each β-diversity distance metric being considered (Table 1). The effect of inoculation source also remained significant when hay extract samples were removed from the analysis. Overall, inoculation source had a higher relative contribution compared to host-genotype in structuring gut bacterial communities. When including the hay extract, 30.6% of the variation in community composition was explained by the inoculation source, while this was reduced to 4.4% when only considering treatments that were inoculated with natural bacterioplankton communities.

Table 2 shows the ten most important taxa in the gut bacterial communities for differentiation between inoculation treatments found by random forest analysis (OOB estimate of error = 15%). These taxa either showed a high variation in mean relative abundance between inoculation treatments (e.g. *Citrobacter* sp. and *Clavibacter* sp.) or were only found in samples of specific inoculation treatments (e.g. *Mycobacterium* and *Rhizobiales*sp.). Taxa identified by DeSeq2 analysis as being differentially abundant between inoculation treatments are listed in Supplementary Table 1.

**Table 2.** Mean relative abundance (±SD) of the ten most important taxa for differentiation between inoculation treatments found by random forest analysis.

Taxon	*Daphnia* pond	Fish pond	Water reservoir	Hay extract
*Citrobacter* sp.	12.6 ± 10.9%	16.6 ± 8.2%	16.3 ± 14.1%	65.6 ± 21.8%
*Polynucleobacter* sp.	1.2 ± 1.1%	5.2 ± 4.3%	1.2 ± 1.6%	0.0 ± 0.1%
*Mycobacterium* sp.	Not present	0.3 ± 0.3%	0.3 ± 0.4%	Not present
Rhizobiales sp.	Not present	0.7 ± 2.4%	0.1 ± 0.2%	1.1% ± 1.1%
*Clavibacter* sp.	1.4 ± 1.3%	0.4 ± 0.4%	0.7 ± 1.4	0.2 ± 0.3%
Rhizobiales sp.	0.0 ± 0.1%	1.1 ± 1.5%	0.2 ± 0.3%	Not present
*Rhodanobacter* sp.	20.6 ± 12.2%	27.4 ± 16.7%	28.0 ± 16.0%	8.4 ± 5.6%
*Limnobacter* sp.	1.1 ± 1.6%	0.6 ± 1.0%	2.9 ± 4.1	0.4 ± 0.5%
Alcaligenaceae sp.	4.7 ± 4.9%	1.1 ± 1.2%	1.0 ± 1.5%	Not present
*Shinella granuli*	0.1 ± 0.4%	2.1% ± 7.9%	0.8 ± 2.4%	0.9 ± 1.1%

For two OTU's, *Polynucleobacter* sp. and *Clavibacter* sp., a positive correlation between their abundance in the medium and in the gut was found (Kendall's test *τ*-test: *p*-adj. < 0.05). This indicates that their colonization rates from the medium influences their abundances in the gut.

### Influence of the host genotype on the gut bacterial community composition

When all inoculation treatments are analyzed together, host genotype had an effect on gut bacterial community composition when differences between samples are measured by the qualitative Unifrac distance (Table 1). When only treatments inoculated with natural bacterioplankton communities are considered, host genotype was found to affect the gut bacterial community composition for both qualitative distance metrics (unweighted Unifrac and Jaccard). Host genotype did not affect gut bacterial diversity or community composition determined by quantitative β-diversity measures when this was assessed over different inoculation treatments.

When the effect of the host genotype on the gut bacterial community composition was assessed separately for each inoculation treatment, it only affected quantitative β-diversity in treatments inoculated with bacterioplankton from the *Daphnia* pond (permutational ANOVA on weighted unifrac; *P* < 0.05) or with the lab-culture derived bacterial communities grown on hay extract (permutational ANOVA on Bray-Curtis dissimilarity; *P* < 0.05). We did not find an effect of the host genotype on the gut bacterial community composition when *Daphnia* were inoculated with bacterioplankton communities from the fish pond or the reservoir.

**Figure 5.**
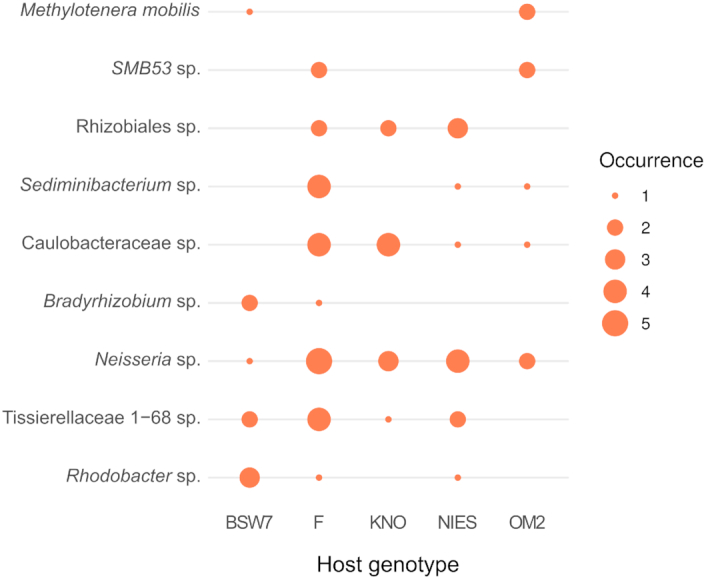
Occurrences of bacterial taxa found to be differentially abundant in host genotypes. Circle size indicates the amount of samples for a specific *Daphnia* genotype that this taxon occurred in. Only taxa that were found to be differentially abundant in less than 20% of permutations are included in this figure.

Figure 5 shows the occurrence patterns of taxa that were identified by DeSeq2 as being significantly differentially abundant between genotypes. Results obtained by random forest analysis were not taken into account because of the high OOB estimate of error (81.7%).

## DISCUSSION

Germ-free *D. magna* belonging to different genotypes were exposed to bacterial communities that originated from either natural bacterioplankton communities or hay extract inoculated with bacteria from *Daphnia* laboratory culture medium. We investigated the influence of the bacterial community to which the experimental animals were exposed, of the experimental animals’ genotype, and of the interaction between both these factors on the gut bacterial community assembly. Our results show that the bacterial community in the medium had a profound effect on the resulting *Daphnia* gut bacterial communities. We further found evidence that the host genotype affected gut bacterial community composition, influencing the occurrence patterns of bacterial taxa over different environments and the relative abundance of bacterial taxa when *Daphnia* were inoculated with bacterial communities from environments where *Daphnia* was known to be present. We did not observe a significant inoculation × host genotype interaction effect, indicating that the effect of medium bacterial communities on the gut bacterial community composition was similar for different genotypes, and that the effect of host genotype was similar in different environments.

Many bacterial taxa were shared between *Daphnia* gut bacterial communities assembled from different environments, indicating that many species that can thrive in the *Daphnia* gut are omnipresent in the environment, but often occurring there at undetectable frequencies. Although in different environments relatively similar gut bacterial communities were established, there was a strong signal of the environmental bacterial community on the *Daphnia* gut bacterial community. For a few taxa (e.g. *Mycobacterium* sp.) we observed that they either occurred in both the medium and the gut or that they were completely absent in both, dependent on inoculation source. Allopatry can generate divergence between gut microbiota due to barriers preventing bacterial dispersal (Moeller *et al*. 2017). Especially in freshwater bacterial metacommunities, dispersal limitation is additionally structured through complex local food web dynamics (Verreydt *et al*. 2012). Our results also show that, to some extent, the *Daphnia* gut bacterial community composition is influenced by local availability of specific bacterial taxa in natural environments. For other taxa (e.g. *Polynucleobacter* sp.) we found a positive correlation between their abundance in the medium and the gut, indicative that the abundance of these taxa in the gut is dependent on inoculation dose. This pattern could be caused by continuous dispersal from the environment into the gut, maintaining larger population sizes under higher dispersal. Also, a lower environmental abundance of a taxon could decrease its initial colonization probability and established bacteria could then further prevent its later colonization (Obadia *et al*. 2017).

A more general trend we observed was that treatments with a higher environmental bacterial diversity resulted in a higher gut bacterial diversity, most likely due to the presence of a larger pool of potential colonizers. Given that gut bacterial diversity was shown to affect *Daphnia* growth rates (Callens *et al*. 2018), it might be worthwhile to investigate if bacterioplankton diversity can affect *Daphnia* population dynamics through its effect on the gut bacterial diversity.

We found a significant effect of the host genotype on qualitative differences between gut bacterial communities on a global scale (i.e when considering the genotype effect over the different bacterial environments). Qualitative differences only account for the presence or absence of taxa, and presence/absence patterns—especially of rare taxa—are often obscured when using quantitative measures that also take relative abundances into account (Lozupone *et al*. 2007). A host genotype effect for presence-absence data, but not for abundance data indicates that differentiation between genotypes over different environments tends to involve relatively rare rather than abundant taxa. Given that the influence of the host genotype on the gut bacterial community composition was more robust for the unweighted unifrac distance, which accounts for the evolutionary distance between taxa, than for the Jaccard distance shows the importance of a phylogenetic component and implies differential occurrences at higher taxonomic ranks. This pattern could possibly be caused by a host genotype specific immune response towards certain bacterial groups, excluding them in some genotypes but not in others (Decaestecker *et al*. 2003). As bacterioplankton community composition in ponds inhabited by *Daphnia* can fluctuate throughout the year, we expect that in natural environments a long-term host genotype effect on the gut bacterial community composition might be similar to what we observe here when considering the genotype effect under a variety of natural bacterioplankton inoculates.

On a local scale (i.e. considering the genotype effect within a single bacterial environment), we only found a significant effect of the host genotype on quantitative measures in two inoculation treatments. Interestingly, both treatments were inoculated with bacterial communities that originated from an environment that contained *Daphnia magna*. These results confirm earlier findings that the *Daphnia* genotype can affect relative abundances of its gut bacterial community members (Macke *et al*. 2017a, Sullam *et al*. 2018; 2020; Sison-Mangus, Metzger and Ebert 2018; Frankel-Bricker *et al*. 2019; Mushegian *et al*. 2019). However, our data show that the environmental pool of bacteria from which *Daphnia* is colonized is an important factor that can influence the presence of this effect. One possible reason for this could be that *Daphnia* has evolved specific responses to bacterial taxa that commonly co-occur with them, producing a host genotype specific regulation of abundances of these taxa.

Although the gut bacterial community was strongly influenced by the bacterial community in the medium, it was also clearly differentiated from it, a finding that is in agreement with previous studies (Freese and Schink 2011; Macke *et al*. 2017, 2020). All gut bacterial communities showed a higher degree of resemblance to each other than to any of the bacterial communities in media inoculated with natural bacterioplankton. This suggests that there is a strong differential selection on the environmental pool of potential colonizers with respect to their establishment success and growth in the *Daphnia* gut. This can reflect environmental filtering driven by abiotic constraints linked to the peculiarities of the gut environment or by properties of the host immune system (Tasiemski *et al*. 2015; Macke *et al*. 2017b) or through competitive exclusion by highly successful taxa in the gut microbiota (Cadotte and Tucker 2017).

There was a strikingly higher degree of resemblance between gut bacterial communities and bacterial communities in the medium of treatments inoculated with hay extract than with the natural assemblages. As the hay extract was inoculated with bacteria from *Daphnia* laboratory culture medium, it appears that it served as a good substrate for those bacteria present in the medium that could also thrive in the *Daphnia* gut. Hay extract is sometimes added to *Daphnia* cultures as a growth promoter. Its specific mode-of-action is unknown, but it might be worthwhile testing whether it exerts its effects through enhancing the growth of beneficial bacteria in the gut of laboratory grown *Daphnia*.

In conclusion, our experiment showed that the composition of the environmental bacterial community has a major influence on the *Daphnia* gut bacterial community. This was partially caused by the presence or absence of specific taxa in the environment, which allows or prevents colonization. Other taxa showed a dose-response relationship, where higher abundances in the environment positively correlated with their abundance in the gut. Finally, we observed that the presence of a host genotype effect on the gut bacterial community composition varied depending on the inoculate, but on a more general scale we observed a consistent effect of host genotype on the occurrence of bacterial taxa in the gut bacterial communities, involving a phylogenetic component. Further research is needed to elucidate the mechanistic basis of both the effect of environmental bacteria and the host genotype on the assembly of *Daphnia* gut bacterial communities. This would require specific experiments designed to address for example the role of priority effects or variation in host immune system genes on the gut bacterial community.

## Supplementary Material

fiaa128_Supplemental_File
