# Supplementary material for: The bacterioplankton community composition and a host genotype dependent occurrence of taxa shape the Daphnia magna gut bacterial community
Source: FEMS Microbiol Ecol. 2020 Jun 23;96(8):fiaa128. doi: 10.1093/femsec/fiaa128 (PMC7360484; doi:10.1093/femsec/fiaa128)
Supplement: fiaa128_Supplemental_File [file fiaa128_supplemental_file.docx]

| **Inoculation source** |  | **Host genotype** |  |
| --- | --- | --- | --- |
| *Cupriavidus* sp. | 40.6 | *Flectobacillus* sp. | 32.7 |
| Cerasicoccaceae sp. | 12.0 | Sphingobacteriaceae sp. | 29.4 |
| Cytophagaceae sp. | 12.0 | *Agrobacterium* sp. | 39.8 |
| Enterobacteriaceae sp. | 0.7 | *Methylotenera mobilis* | 18.1 |
| Sphingomonadales sp. | 16.7 | *Leadbetterella* sp. | 39.3 |
| *Limnobacter* sp. | 12.1 | *Methylibium* sp. | 50.7 |
| *Flavobacterium* sp. | 12.0 | Alcaligenaceae sp. | 53.8 |
| Caulobacteraceae sp. | 8.6 | *Anaerococcus* sp. | 50.7 |
| *Paracoccus* sp. | 3.0 | *Rhodobacter* sp. | 8.0 |
| *Brevundimonas diminuta* | 15.8 | *Methylobacterium* sp. | 39.0 |
| *Methylotenera mobilis* | 33.1 | *Flavobacterium* sp. | 30.8 |
| *Yersinia* sp. | 6.1 | *Staphylococcus* sp. | 41.8 |
| Alcaligenaceae sp. | 49.9 | 1-68 sp. | 14.8 |
| *Anaerococcus* sp. | 54.4 | *Neisseria* sp. | 19.7 |
| Rhizobiaceae sp. | 37.6 | *Bradyrhizobium* sp. | 6.4 |
| *Rhodoferax* sp. | 21.3 | Caulobacteraceae sp. | 17.6 |
| *Polynucleobacter* sp. | 30.5 | *Sediminibacterium* sp. | 7.5 |
| *Methylobacterium* sp. | 25.0 | Rhizobiales sp. | 17.5 |
| *Pantoea agglomerans* | 29.6 | SMB53 sp. | 13.7 |
| *Legionella* sp. | 42.6 | *Psychromonas* sp. | 26.5 |
| *Mycobacterium* sp. | 12.0 |  |  |
| *Anaerococcus* sp. | 38.7 |  |  |
| *Staphylococcus* sp. | 45.0 |  |  |
| *Neisseria subflava* | 24.2 |  |  |
| *Stenotrophomonas maltophilia* | 13.5 |  |  |
| Verrucomicrobiaceae sp. | 13.5 |  |  |
| *Flavobacterium succinicans* | 11.8 |  |  |
| *Arthrobacter* sp. | 12.7 |  |  |
| *Cellvibrio sp.* | 12.9 |  |  |
| *Phycicoccus* sp. | 7.7 |  |  |
| *Mycobacterium* sp. | 3.8 |  |  |

**Supplementary table 1:** Bacterial taxa identified by DeSeq2 to be differentially abundant between inoculation treatments (left columns) or host genotypes (right columns). The number next to each taxon indicates the percentage of random permutations this taxon was found to be differentially abundant in, higher values indicate that a significant differential abundance might be caused by a large number of zero values.
